# Proteomic Changes of Porcine Oocytes After Vitrification and Subsequent *in vitro* Maturation: A Tandem Mass Tag-Based Quantitative Analysis

**DOI:** 10.3389/fcell.2020.614577

**Published:** 2020-12-23

**Authors:** Baoyu Jia, Decai Xiang, Xiangwei Fu, Qingyong Shao, Qionghua Hong, Guobo Quan, Guoquan Wu

**Affiliations:** ^1^College of Veterinary Medicine, Yunnan Agricultural University, Kunming, China; ^2^Yunnan Provincial Engineering Laboratory of Animal Genetic Resource Conservation and Germplasm Enhancement, Yunnan Animal Science and Veterinary Institute, Kunming, China; ^3^College of Animal Science and Technology, China Agricultural University, Beijing, China

**Keywords:** pig, vitrification, oocytes, proteome, TMT, PRM

## Abstract

Cryopreservation of immature germinal vesicle (GV) oocytes is a promising strategy in pigs but still results in reduced oocyte quality due to inevitable cryodamages. Recently, there has been more focus on the molecular changes of oocytes after vitrification, but the alteration in the proteome level remains elusive. The aim of this study therefore was to decipher the proteomic characteristics of porcine GV oocytes following vitrification and *in vitro* maturation (IVM) by using tandem mass tag (TMT)-based quantitative approach and bioinformatics analysis. A total of 4,499 proteins were identified, out of which 153 presented significant difference. There were 94 up-regulated and 59 down-regulated proteins expressed differentially in the vitrified oocytes. Functional classification and enrichment analyses revealed that many of these proteins were involved in metabolism, signal transduction, response to stimulus, immune response, complement, coagulation cascades, and so on. Moreover, a parallel reaction monitoring technique validated the reliability of TMT data through quantitative analysis for 10 candidate proteins. In conclusion, our results provided a novel perspective of proteomics to comprehend the quality change in the vitrified porcine GV oocytes after IVM.

## Introduction

Cryopreservation of gametes and embryos as an important biotechnological tool has been applied extensively in gene bank collections, animal breeding, and human-assisted reproductive technologies (Zhou and Li, 2009; Robles et al., 2019). In pigs, vitrification is the most common method used to cryopreserve oocytes and embryos (Saragusty and Arav, 2011; Mandawala et al., 2016). It has been confirmed that porcine oocytes vitrified at the immature germinal vesicle (GV) stage have a normal ability of nuclear maturation and *in vitro* fertilization, resulting in live offspring after embryo transfer (Somfai et al., 2014). With the continuous improvement of their cryosurvival rate (Wu et al., 2017; Appeltant et al., 2018), porcine GV oocytes seem to be more suitable for vitrification. However, the blastocyst yield of vitrified oocytes is still very low as compared with fresh oocytes, regardless of whatever strategy for *in vitro* embryo production is chosen (Fujihira et al., 2004; Nohalez et al., 2015; Wu et al., 2017; Casillas et al., 2018). This implies that vitrification may produce a certain degree of sublethal damages in the oocytes, thus hindering subsequent embryo development.

The high concentration of cryoprotectants and rapid cooling rate into liquid nitrogen (LN_2_) are required for successful vitrification (Arav, 2014). Meanwhile, there are some disadvantageous factors in the process of vitrification, including strong physical changes of temperature and osmolarity, and chemical stress caused by pH variation and cryoprotectant toxicity (Szymañska et al., 2019). These abnormal physiological conditions inevitably lead to structural and functional damages of various mammalian oocytes after vitrification, such as cytoskeleton disruption (Egerszegi et al., 2013), chromosomal disorder (Tamura et al., 2013), organelle dysfunction (Fu et al., 2009; Lowther et al., 2009; Jang et al., 2014), oxidative stress (Nohales-Córcoles et al., 2016), calcium disturbance (Wang et al., 2017a), apoptosis (Niu et al., 2016), and epigenetic alteration (Chen et al., 2019). In recent years, increasing concern is focused on the molecular changes in oocytes induced by the vitrification. Therefore, the global analysis strategies of transcriptome, proteome, and metabolome are becoming ever more valuable in this research field. Several studies have utilized the RNA sequencing (RNA-seq) technique to analyze mRNA transcriptome of the vitrified oocytes in mice (Gao et al., 2017), cattle (Wang et al., 2017b; Huang et al., 2018; Zhang et al., 2019), and pigs (Jia et al., 2019). These data obtained from RNA-seq greatly help researchers to understand the oocyte cryodamages. Nevertheless, transcriptomic analysis may not be comprehensive, because numerous cellular stress responses involve changes at the protein level (Miura and Endo, 2010). It has been confirmed that proteomic analysis is more directly related to the cellular function than gene and transcript analyses (Tyanova et al., 2016). Up to now, there is no report about the proteomic changes of vitrified oocytes.

The proteomics as a post-genomic biotechnology can comprehensively analyze the entire proteins present in protein sample including the information on their abundances, structure, modification, and regulatory networks (Pischetsrieder and Baeuerlein, 2009; Liao et al., 2015). Mass spectrometry (MS) is the most widely used technique in proteomics to identify and quantify proteins (Ma et al., 2011). Tandem mass tag (TMT) labeling coupled with liquid chromatography (LC) tandem MS (MS/MS) (LC-MS/MS), a new developed quantitative proteomic technique, has gained popularity in various research fields because of its multiple advantages (Pagel et al., 2015; Guo et al., 2019). On the other hand, the parallel reaction monitoring (PRM) technique with higher sensitivity and specificity can detect and quantify the target proteins (Peterson et al., 2012; Urisman et al., 2017). Therefore, the aim of this study was to obtain the proteomic profile in vitrified porcine GV oocytes after *in vitro* maturation (IVM) along with the underlying molecular mechanisms, using the TMT-based method and bioinformatics analysis. Moreover, we also performed a PRM assay to validate the proteins selected from TMT proteomic data.

## Materials and Methods

All chemicals were purchased from Sigma-Aldrich Chemical Company (St. Louis, MO, United States), unless otherwise specified.

### Oocyte Collection and Grouping

Porcine ovaries were obtained from pre-pubertal crossbred Landrace gilts at a local abattoir and transported to the laboratory within 2 h in saline supplemented with 75 mg/L of penicillin G potassium and 50 mg/L of streptomycin sulfate at 35–37°C. Follicular contents were aspirated from antral follicles (3–8 mm in diameter) using a disposable syringe with 18-gauge needle. The sediments containing cumulus oocyte complexes (COCs) were washed twice in Tyrode’s lactate-HEPES-polyvinyl alcohol (TLH-PVA) medium (Funahashi et al., 1997). COCs were selected under a stereomicroscope (Olympus, Tokyo, Japan), and those with dense cumulus cells (CCs) and uniform cytoplasm were used in the experiments.

In each experimental batch for oocyte collection, about three-fifths of the obtained COCs were vitrified, warmed, and then cultured for maturation, and the remaining COCs were directly subjected to IVM as the control. After IVM, mature metaphase II (MII) oocytes (first polar body extrusion) from these two groups were collected for the following proteomic experiments.

### Oocyte Vitrification and Warming

Porcine GV oocytes were subjected to vitrification and warming in the form of COCs, according to a previous report (Wu et al., 2017). All solutions were prepared using a base medium (BM), which was Dulbecco’s phosphate-buffered saline (DPBS; Gibco, Grand Island, NY, United States) supplemented with 20% (v/v) synthetic serum substitute (Irvine Scientific, Santa Ana, CA, United States). First, COCs were put into BM for 3 min and then equilibrated with 5% (v/v) ethylene glycol (EG) for 10 min at 25°C. Subsequently, 10–15 COCs for each group were exposed to vitrification solution (VS) consisting of BM supplemented with 0.6 M sucrose, 50 mg/ml of polyvinylpyrrolidone and 35% (v/v) EG. After 20–30 s at 25°C, these COCs were loaded onto a Cryotop carrier (Kitazato Biopharma, Shizuoka, Japan) with minimum volume of VS and immediately plunged into LN_2_.

Warming manipulation was performed on a 42°C hot plate, and warming solutions were also pre-heated to 42°C. For warming, the tip of Cryotop was dipped into 1.0 M of sucrose for 1 min. The vitrified COCs were picked out and transferred stepwise into 0.5 and 0.25 M of sucrose for 2.5 min, respectively. Finally, they were incubated in BM for 5 min and then submitted to IVM.

### Oocyte *in vitro* Maturation

For IVM, about 50–70 COCs were cultured in each well of a 24-well plate (Costar, Corning, NY, United States) containing 500 μl of IVM medium covered by mineral oil for 42–44 h at 39°C in an atmosphere of 5% CO_2_ with saturated humidity. The IVM medium was TCM-199 (Gibco, Grand Island, NY, United States) supplemented with 3.05 mM of D-glucose, 0.57 mM of cysteine, 0.91 mM of sodium pyruvate, 10% (v/v) porcine follicular fluid, 10 ng/ml of epidermal growth factor, and 0.5 μg/ml of each follicle-stimulating hormone and luteinizing hormone.

With regard to the vitrified COCs, their survival was evaluated after 2 h of IVM culture based on morphological characteristics under a stereomicroscope. Oocytes with disappeared vitelline membrane and/or altered cytoplasm were considered dead and removed, with surviving COCs continued to IVM.

At the end of IVM, both fresh and vitrified oocytes were gently denuded of CCs by repeated pipetting in TLH-PVA medium supplemented with 0.1% (w/v) hyaluronidase. Only MII oocytes with evenly granular cytoplasm were selected and then stored at –80°C to provisionally conserve. When the total number of collected oocytes was enough, they were pooled based on each sample requirement and then prepared for TMT and PRM analyses.

### Protein Extraction, Digestion, and Tandem Mass Tag Labeling

Three biological replicates were performed, and approximately 1,500 oocytes were used for each sample. For protein extraction, all samples were lysed with lysis buffer (8 M of urea, 1% Protease Inhibitor Cocktail) on ice using a high-intensity ultrasonic processor, in order to obtain the supernatant following centrifugation at 12,000 *g* at 4°C for 10 min. Protein concentration was determined using Bicinchoninic Acid Protein Assay Kit (Pierce, Rockford, IL, United States), according to the manufacturer’s instructions.

For digestion, protein solution was reduced with 5 mM of dithiothreitol (final concentration) for 30 min at 56°C, alkylated with 11 mM of iodoacetamide for 15 min in darkness at room temperature, and then diluted to urea concentration of less than 2 M. The trypsin at a mass ratio of 1:50 (trypsin:protein) at 37°C overnight was used to the first digestion and continued for a post-digestion with 1:100 mass ratio (trypsin:protein) for 4 h.

After trypsin digestion, peptides were desalted by Strata X C18 SPE column (Phenomenex, Torrance, CA, United States), vacuum-dried, and then reconstituted in 0.5 M of triethylammonium bicarbonate (TEAB). For TMT labeling, one unit of TMT reagent (Thermo Fisher Scientific) was thawed, reconstituted in acetonitrile, and mixed with peptides for 2 h at room temperature. Then the peptide mixtures were pooled, desalted, and dried by vacuum centrifugation.

### High-Performance Liquid Chromatography Fractionation and Liquid Chromatography–Tandem Mass Spectrometry Analysis

High pH reversed-phase high-performance LC (HPLC) was performed to fractionate the labeled peptides, using an Agilent 300 Extend C18 column (5-μm particles, 4.6-mm i.d., 250-mm length; Agilent, Santa Clara, CA, United States). Briefly, tryptic peptides were first separated with a gradient of 8 to 32% acetonitrile (pH 9.0) over 60 min into 60 fractions, combined into nine fractions, and then vacuum-dried. Subsequently, the peptides were dissolved in 0.1% formic acid (solvent A) and directly loaded onto a homemade reversed-phase analytical column (15-cm length, 75-μm i.d.) to elute with gradient solvent B (0.1% formic acid in 98% acetonitrile). A linear gradient of solvent B was used as follows: 7 to 16% over 50 min, 16 to 30% in 35 min, 30 to 80% in 2 min, and 80% for the last 3 min. Flow rate was 400 nl/min on an EASY-nLC 1000 ultraperformance LC (UPLC) system (Thermo Fisher Scientific, Waltham, MA, United States).

The peptides were subjected to nanospray ionization with a voltage of 2.0 kV and then detected by MS/MS in Q Exactive Plus (Thermo Fisher Scientific, Waltham, MA, United States) coupled online to the UPLC system. MS and MS/MS spectra were acquired in the Orbitrap with 60,000 resolution at 350–1,550 *m*/*z* and 30,000 resolution at 100 *m*/*z*, respectively. A data-dependent acquisition was performed with the following parameters: each MS scan followed by 20 MS/MS scans with 30.0-s dynamic exclusion. After MS scan, the 10 most abundant precursor ions were selected for higher-energy collisional dissociation (HCD) fragmentation with a normalized collision energy (NCE) setting of 32%. Automatic gain control (AGC) and maximum injection time (max IT) were set at 5E4 and 70 ms, respectively.

### Database Search and Bioinformatics Analysis

The resulting MS/MS data were processed using Maxquant search engine (v1.5.2.8) against the *Sus scrofa* UniProt proteome database (40,708 sequences) concatenated with reverse decoy database. The parameters were set as follows: (1) trypsin/P was specified as the cleavage enzyme; (2) two missing cleavages were allowed; (3) the minimum peptide length was seven amino acids; (4) the maximum number of modifications per peptide was 5; (5) the mass tolerance for precursor ions was 20 ppm in the first search and 5 ppm in the main search; (6) fragment ion mass tolerance was 0.02 Da; (7) carbamidomethylation on cysteine was fixed modification; (8) oxidation on methionine and N-terminal acetylation were variable modification; and (9) false discovery rate was adjusted to <1%. Student’s *t*-test was used to evaluate the significant differences. The proteins with fold change of ≥1.20 or ≤0.83 and *p*-value < 0.05 were considered as differentially expressed proteins (DEPs) on the basis of the related TMT or iTRAQ studies.

Protein Gene Ontology (GO) annotation was derived from the UniProt-GOA database^1^ according to biological process, cellular component, and molecular function (Barrell et al., 2009). If proteins are not annotated by this database, the InterProScan^2^ software was used to annotate GO function based on protein sequence alignment. The protein subcellular localization was predicted by Wolfpsort^3^ (Horton et al., 2007). We used an online service tool KAAS^4^ to annotate Kyoto Encyclopedia of Genes and Genomes (KEGG) database descriptions (Kanehisa et al., 2017). Subsequently, all proteins were mapped to corresponding pathways in the database using KEGG mapper^5^. As a result, a two-tailed Fisher’s exact test was employed to test the enrichment of the DEPs against all identified proteins. GO terms and KEGG pathways with a corrected *p*-value < 0.05 were considered to be significantly enriched. Protein–protein interaction (PPI) network was analyzed by STRING 11.0^6^ and then visualized in Cytoscape software (version 3.7.2).

### Parallel Reaction Monitoring Validation

Only proteins identified with high confidence peptide sequence were selected for PRM validation based on the TMT data. Three biological replicates were included, each of which was performed with around 1,000 oocytes. First, tryptic digested peptides through the TMT method described above were dissolved in solvent A and eluted using a homemade reversed-phase analytical column with gradient solvent B (6 to 25% over 40 min, 25 to 35% in 12 min, climbing to 80% in 4 min, and holding at 80% for the last 4 min) at 500 nl/min of flow rate. Then, the eluted peptides were analyzed using nanospray ionization source and Q Exactive Plus coupled online to the UPLC. An electrospray voltage of 2.2 kV was applied. By the Orbitrap, full MS was detected at a resolution of 70,000 with 350–1,060 *m*/*z* scan range (AGC, 3E6; max IT, 50 ms), followed by 20 MS/MS scans at a resolution of 17,500 (AGC, 1E5; max IT, 120 ms; isolation window, 1.6 *m*/*z*) in a data-independent acquisition. Precursor ions were fragmented through HCD with an NCE of 27. The PRM data were processed using Skyline software (version 3.6, MacCoss Lab, University of Washington, United States) (MacLean et al., 2010). The results for each peptide were quantified according to the fragment ion peak area from its corresponding transitions, and statistical significance was set at *p*-value < 0.05 using Student’s *t*-test.

## Results

### Overview of Tandem Mass Tag-Based Proteomic Data

In the present study, the survival rates of vitrified GV oocytes after both 2 h of warming and IVM were 86.8 and 83.1%, respectively; the fresh GV oocytes had 93.5% survival following IVM. Moreover, the MII rate was 85.9% for vitrified oocytes and 88.6% for fresh oocytes.

**FIGURE 1:**
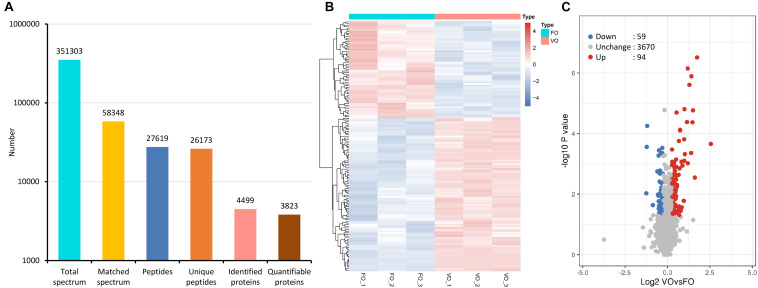
Tandem mass tag (TMT)-based quantitative proteomic sequencing results. **(A)** Summary of tandem mass spectrometry database search analysis. **(B)** A heatmap with hierarchical clustering of differentially expressed proteins (DEPs). In the heatmap, the expression of DEPs in different samples is shown with different colors, with red indicating high expression and blue indicating low expression. Each line represents a protein, and each column represents a group of samples. **(C)** A volcano plot of DEPs. Proteins with fold change of ≥1.20 or ≤0.83 and *p*-value < 0.05 were considered statistically significant. Red dots indicate significant up-regulated proteins, blue dots indicate significant down-regulated proteins, and gray dots indicate proteins without differences. The *X*-axis represents fold change, and *Y*-axis means *p*-value. FO, fresh oocytes; VO, vitrified oocytes.

First of all, biological replicates were validated by the relative standard deviation distribution, which displayed the precision and reproducibility of our proteomic datasets (Supplementary Figure 1A). By a strict quality control, we obtained a total of 3,51,303 spectra (58,348 matched), and 27,619 peptides (26,173 unique peptides) were detected from among them (Figure 1A). Moreover, the average mass error of peptides was less than 6 ppm, indicating a high mass accuracy of the MS data as requirement during the processes (Supplementary Figure 1B). Further analysis showed that the lengths of most peptides were distributed at the range of 7 to 20 amino acids, which meant reliable results (Supplementary Figure 1C). Finally, 4,499 proteins were identified, 3,823 of which were quantified (Figure 1A). The detailed protein information is provided in Supplementary Table 1.

Following statistical analysis, 153 proteins with fold-change ≥1.20 or ≤0.83 and *p*-value < 0.05 were considered as the DEPs. Among them, a total of 94 up-regulated and 59 down-regulated DEPs were identified (Supplementary Table 2). In addition, a heatmap represented a hierarchical cluster of the DEPs, and a volcano plot also indicated average changes of individual protein abundance (Figures 1B,C).

### Functional Classification Analysis

**FIGURE 2:**
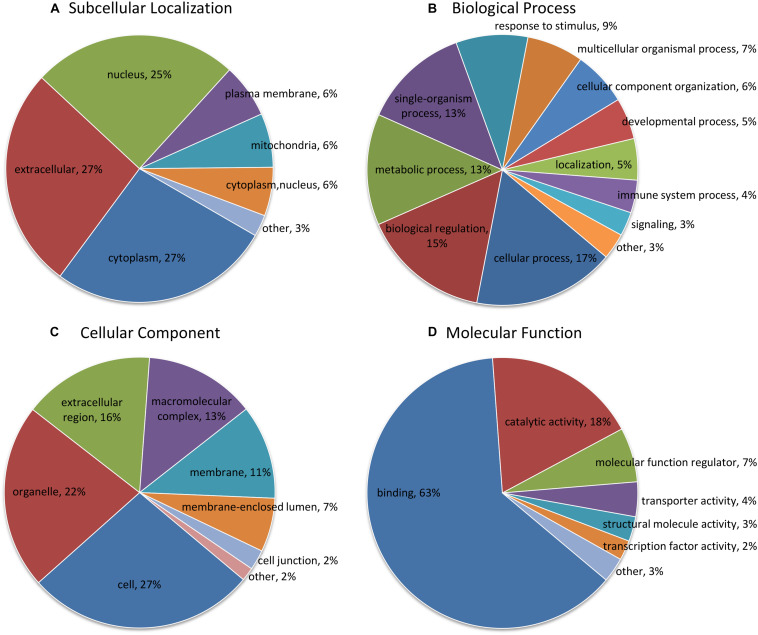
Functional classification analysis of differentially expressed proteins (DEPs). **(A)** Subcellular localization prediction; **(B)** Gene Ontology (GO) classification in terms of biological process; **(C)** GO classification in terms of cellular component; **(D)** GO classification in terms of molecular function. The term and proportion of DEPs are shown in each sector.

**FIGURE 3:**
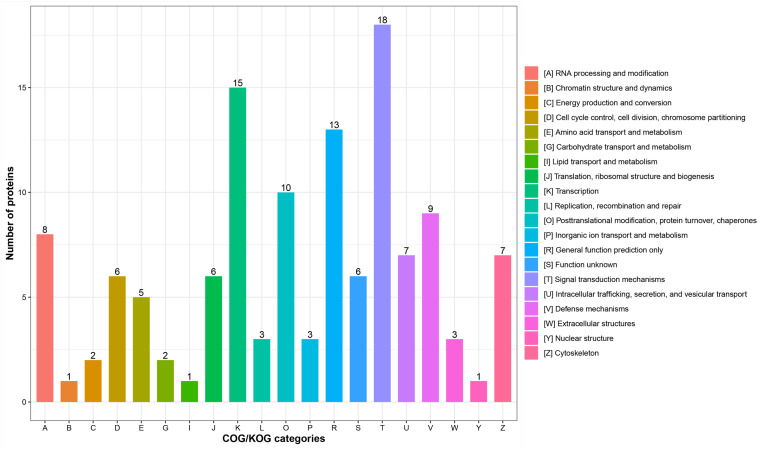
Clusters of Orthologous Groups of protein/EuKaryotic Orthologous Groups (COG/KOG) functional classification analysis of differentially expressed proteins (DEPs). The DEPs were aligned to COG/KOG database and classified into 20 functional clusters. Each bar represents the mummer of DEPs.

According to subcellular localization predictions, these DEPs were mainly distributed in the “cytoplasm” (27%), “extracellular” (27%), “nucleus” (25%), “plasma membrane” (6%), and “mitochondria” (6%) (Figure 2A and Supplementary Table 3). Next, GO functional classification was performed for the DEPs. We found that these DEPs were cataloged in 27 GO terms, including 12 biological process, 8 cellular component, and 7 molecular function (Figures 2B–D and Supplementary Table 4). More detail, the top five GO terms in biological process consisted of “cellular process” (17%), “biological regulation” (15%), “metabolic process” (13%), “single-organism process” (13%), and “response to stimulus” (9%). The cellular component results showed that “cell” (27%), “organelle” (22%), “extracellular region” (16%), “macromolecular complex” (13%), “membrane” (11%), “membrane-enclosed lumen” (7%), and “cell junction” (2%) were mostly classifications. Moreover, “binding” (63%) and “catalytic activity” (18%) were the two most prominent terms in the molecular function. On the other hand, the functional classification of DEPs was also predicted by performing Clusters of Orthologous Groups of protein/EuKaryotic Orthologous Groups (COG/KOG) analysis. As shown in Figure 3 and Supplementary Table 5, these DEPs were assigned to 20 COG/KOG categories, and the largest category was “signal transduction mechanisms,” followed by “transcription,” “general function prediction only,” “post-translational modification, protein turnover, chaperones,” “defense mechanisms,” and so on.

### Functional Enrichment Analysis

**FIGURE 4:**
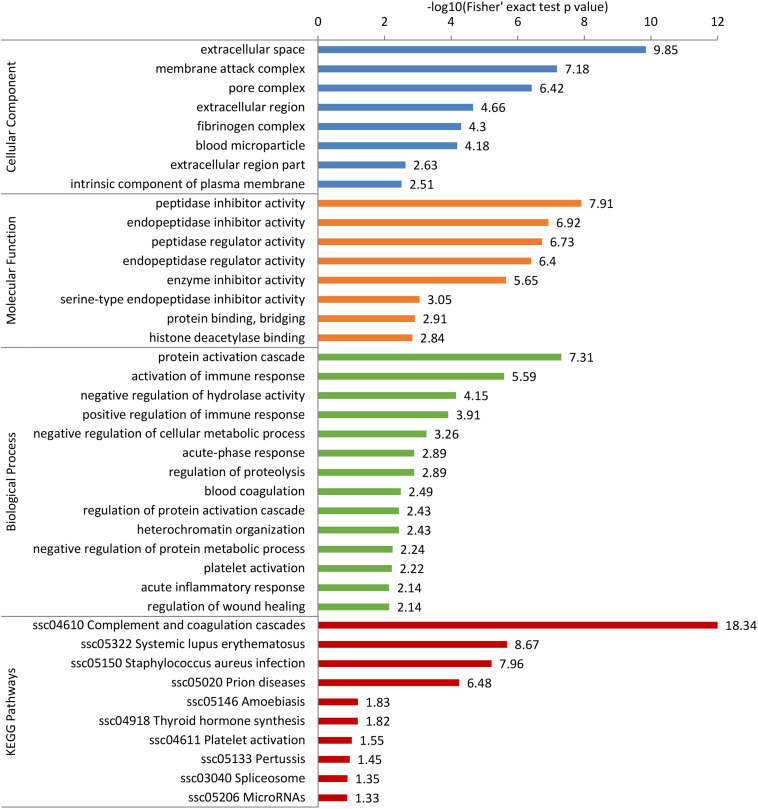
Gene Ontology (GO) and Kyoto Encyclopedia of Genes and Genomes (KEGG) pathway enrichment analysis of differentially expressed proteins (DEPs). The DEPs were enriched into cellular component, molecular function, biological process, and KEGG pathway. The bar represents the enrichment score associated with each term, and score value is shown as -log10 (Fisher’s exact test *p*-value).

To further predict the possible roles of DEPs, functional enrichment analysis was conducted according to GO annotation and KEGG pathway. First, detailed information of GO term enrichment is shown in Figure 4 and Supplementary Table 6. Within the cellular component, the enriched terms were “extracellular space,” “membrane attack complex,” “pore complex,” “extracellular region,” etc. Molecular function enrichment indicated peptidase and endopeptidase inhibitor/regulator activity. Regarding biological process, the top five terms included “protein activation cascade,” “activation of immune response,” “negative regulation of hydrolase activity,” “positive regulation of immune response,” and “negative regulation of cellular metabolic process.” In addition, the KEGG enrichment analysis found several main pathways such as “complement and coagulation cascades,” “thyroid hormone synthesis,” and “spliceosome” (Figure 4 and Supplementary Table 7).

### Protein–Protein Interaction Network

**FIGURE 5:**
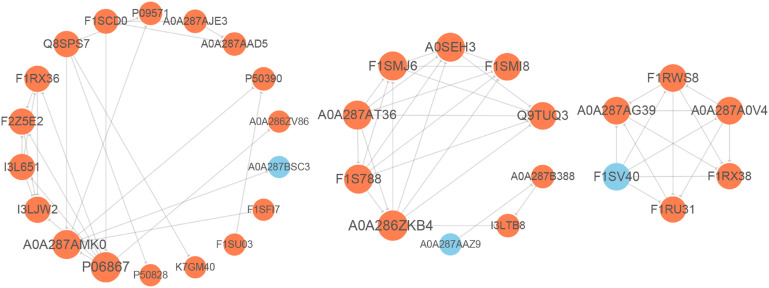
Protein–protein interaction (PPI) network analysis of differentially expressed proteins (DEPs). The gray lines represent direct interactions between two proteins. The circles stand for DEPs, orange circles indicate up-regulated proteins, and blue circles indicate down-regulated proteins.

For elucidating the functional interactions of DEPs, we performed a PPI network analysis among DEPs. As shown in Figure 5, three highly interconnected clusters were identified in this PPI network. There were seven DEPs with a high degree of connectivity, including plasminogen (PLG, P06867), complement C5a anaphylatoxin (C5, A0A286ZKB4), complement component C9 precursor (C9, F1SMJ6), serum albumin (ALB, A0A287AMK0), complement component C8 beta chain precursor (C8B, A0A287AT36), complement component C8 gamma chain precursor (C8G, A0SEH3), and complement C8 alpha chain (C8A, F1S788); and most of them participated in complement component. In addition, pleiotropic regulator 1 (PLRG1, F1RX38), thioredoxin like 4A (TXNL4A, A0A287A0V4), U4/U6.U5 tri-snRNP-associated protein 1 (SART1, F1RU31), small nuclear ribonucleoprotein polypeptide A’ (SNRPA1, F1RWS8), and splicing factor 3a subunit 3 (SF3A3, F1SV40) constructed an interconnected cluster and were involved in the “spliceosome” pathway.

### Parallel Reaction Monitoring Validation

**FIGURE 6:**
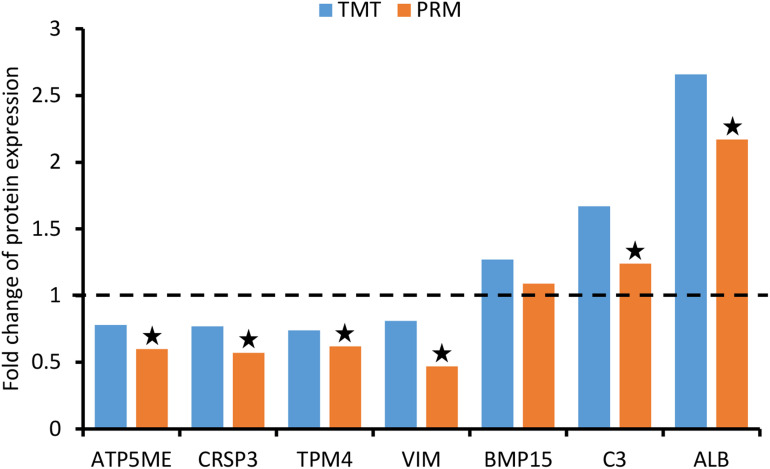
Validation of differentially expressed proteins (DEPs) using parallel reaction monitoring (PRM) analysis. There are seven candidate DEPs obtained to verify the tandem mass tag (TMT) results.

To validate the TMT results, a PRM analysis was performed to detect the expression of 10 candidate DEPs. Due to the requirements of protein characteristics and abundance, we obtained the abundant values of seven proteins by quantitative data of target peptide fragments, including ATP synthase subunit e (ATP5ME, Q9MYT8), calcitonin receptor-stimulating peptide 3 (CRSP3, A0A286ZNZ6), tropomyosin alpha-4 chain (TPM4, P67937), vimentin (VIM, P02543), bone morphogenetic protein 15 (BMP15, F1RV73), complement C3 (C3, I3LTB8), and serum albumin (ALB, A0A287AMK0). As shown in Figure 6, these proteins were exactly the same trend as quantified using TMT method, although the fold change varied between the two techniques. Besides BMP15, they showed significant differences between groups.

## Discussion

In the process of vitrification and warming, various damages may occur in the oocytes, which is reflected in their death or subsequent lower embryo developmental competence. The cryoinjury of oocytes is a complex problem requiring multifaceted solutions and has not yet been not fully elucidated. Moreover, very little information is available about changes at the molecular level of oocytes after vitrification. In the present study, a TMT-based quantitative proteomics in combination with bioinformatics analysis was successfully performed for the vitrified porcine GV oocytes following IVM, which could help to understand the effect of vitrification on oocyte quality from a proteomic perspective. On the other hand, we also verified the accuracy and reliability of TMT proteomic data by a PRM analysis, this technique is capable of quantifying multiple proteins simultaneously.

A large number of studies have confirmed that vitrification of oocytes causes mitochondrial dysfunction, including abnormal distribution, broken structure, lower membrane potential, insufficient ATP production, and other issues (Ito et al., 2020). In the present study, we found 10 DEPs located in mitochondria according to the subcellular localization analysis. Among these, ATP5ME and ATP synthase F(0) complex subunit C3 (ATP5MC3, F1RZI0) classified to “energy production and conversion” in COG/KOG categories could be responsible for the abnormality in mitochondrial ATP synthesis of vitrified oocytes. Mitochondrial calcium uptake 2 (MICU2, A0A287AZY0) as a high confidence mitochondrial-localized protein is the genuine gatekeeper of mitochondrial calcium uniporter (Patron et al., 2014), and its knockdown induces a persistent increase in mitochondrial calcium uptake (Matesanz-Isabel et al., 2016). Vitrification is reported to increase the mitochondrial calcium level in bovine oocytes (Wang et al., 2017a), which may be related to lower expression level of MICU2 protein according to our findings.

In the GO classification analysis, we focused on the “response to stimulus” in biological process, due to the vitrification as a stress factor, and we found 33 DEPs were classified to this term. Among of the proteins, DNA-(apurinic or apyrimidinic site) lyase (APEX1, A0A287BTC2) plays a primary role in base excision repair. It is reported that vitrification of oocytes induces DNA damage (Kopeika et al., 2014). From the present study, the decreased level of APEX1 protein could be at least partially contributed to the increased DNA damage in vitrified oocytes. Based on molecular function classification, “binding” term accounted for most of the DEPs (up to 106 proteins), suggesting that vitrification primarily affected the protein binding activity of oocytes.

Gene Ontology enrichment analysis revealed that “histone deacetylase binding” term was significantly up-regulated in the molecular function, enriched DEPs including sin3 histone deacetylase corepressor complex component (SUDS3, F1RKH0), DEAD-box helicase 20 (DDX20, F1SBP3) and geminin, and DNA replication inhibitor (GMNN, F1RUD8). These three proteins are required for histone deacetylase activity and involved in early embryonic development (Mouillet et al., 2008; Yang et al., 2011; Zhang et al., 2013). The histone acetylation is important for the maturation of porcine oocytes. Inhibition of histone deacetylase has been reported to affect oocyte maturation and embryonic development (Jin et al., 2014). So histone deacetylase in the vitrified oocytes may increase due to overproduction of the above-mentioned proteins, resulting in reduced oocyte quality. Moreover, the molecular function was also enriched with terms related to “peptidase inhibitor activity,” “endopeptidase inhibitor activity,” and “enzyme inhibitor activity,” suggesting the lack of protease activity in the vitrified oocytes. On the other hand, we also found the enriched terms of “negative regulation of cellular metabolic process” and “negative regulation of protein metabolic process” in the biological process, which is not beneficial to the process of cell metabolism for vitrified oocytes. As we have known, the metabolic activity is essential for IVM of mammalian oocytes. For example, cysteine is usually added to IVM medium to promote the maturation capacity of porcine oocytes (Sawai et al., 1997). However, cystatin (CST3, Q0Z8R0) is a natural inhibitor of cysteine peptidases; its increased expression in vitrified oocytes may result in the inhibition of cysteine metabolic enzyme and has an adverse impact on the oocyte maturation.

The homeostasis of complement system (an innate immune system) is regulated strictly, avoiding insufficient or excessive activation (Cheng et al., 2018). In our study, the KEGG pathway analysis showed that 16 DEPs were enriched in the “complement and coagulation cascades” (ssc04610) accounting for the largest part, and most of these were complement component proteins. Moreover, there were five up-regulated proteins with a fold change >2 involved in the “membrane attack complex” term (GO:0005579). The results suggested that the complement system in oocytes might be activated by vitrification to play a role in the cryodamages. In addition, some of the above proteins were identified as hub proteins in the PPI network, and also several were enriched in “activation of immune response” and “positive regulation of immune response” terms that belonged to the GO biological process. Therefore, further studies need to confirm whether the degree of immune response is beneficial or deleterious to oocytes after vitrification. On the other hand, we found that the “spliceosome” pathway (ssc03040) enriched six up-regulated DEPs out of which five formed a PPI cluster. Spliceosome is primarily responsible for removing non-coding introns from pre-mRNA (Karamysheva et al., 2015). The above results might indicate higher spliceosome assembly and activation, which is possibly associated with abnormal mRNA maturation and gene expression in vitrified oocytes.

Interestingly, the present study helped to find an important signaling pathway of hormone synthesis in vitrified oocytes: “thyroid hormone synthesis” pathway (ssc04918), containing transthyretin (TTR, P50390), and other two proteins. Several studies confirm that thyroid hormones have positive effect on cultured oocytes and/or their supporting cells (Costa et al., 2013). In our previous study, the RNA-seq results showed a decreased expression of *THRA* gene in CCs derived from vitrified porcine GV oocytes after IVM (Jia et al., 2019). Therefore, vitrification is likely to disrupt the normal thyroid hormone action in oocytes and their CCs during IVM. The next attempt is to investigate whether the exogenous thyroid hormones including triiodothyronine and thyroxine will be able to improve the maturation quality of vitrified oocytes and the relevant mechanisms. On the other hand, it is reported that treatment of oocytes with proteasome inhibitor MG132 during IVM can modulate the protein expression and subsequent embryonic development (You et al., 2012). So we also will make further improvements in IVM strategy to mitigate the proteomic changes of vitrified oocytes, for instance, the regulation of transcription through chemical reagent treatment. Moreover, this study is not exempt of limitations. Future research needs us to carry out the functional exploration for key proteins by utilizing some techniques such as small interfering RNA knockdown and protein overexpression, in order to elucidate their role in the vitrified oocytes.

## Conclusion

In conclusion, the present study provided, for the first time to our knowledge, comprehensive proteomic information of porcine oocytes after vitrification and subsequent IVM, identifying a total of 94 up-regulated and 59 down-regulated DEPs. Moreover, bioinformatics analysis revealed that vitrification of oocytes caused alterations in metabolism, signal transduction, stress response, complement activation, immune, and other cell functions. All these findings can largely enrich the study on oocyte cryodamages and provide a novel perspective to comprehend the maturation status of vitrified GV oocytes.

## Data Availability Statement

The raw data supporting the conclusions of this article will be made available by the ProteomeXchange with identifier PXD023107.

## Author Contributions

GW and BJ conceived the experiments. BJ, DX, QS, QH, and GW conducted the experiments. BJ, DX, and XF performed statistical analysis and figure generation. GW, BJ, and DX wrote the manuscript. XF and GQ reviewed the manuscript. All authors have read and agreed to the published version of the manuscript.

## Conflict of Interest

The authors declare that the research was conducted in the absence of any commercial or financial relationships that could be construed as a potential conflict of interest.

## Footnotes

1 http://www.ebi.ac.uk/GOA/

2 http://www.ebi.ac.uk/interpro/

3 http://www.genscript.com/psort/wolf_psort.html

4 http://www.genome.jp/kaas-bin/kaas_main

5 https://www.genome.jp/kegg/mapper.html

6 https://string-db.org/

## Supplementary Material

Supplementary Figure 1 Quantitative proteome analysis and quality control validation of mass spectrometry data. **(A)** Relative standard deviation (RSD) distribution of repeated samples; **(B)** Average peptide mass error; **(C)** Length distribution of all identified peptides.

Supplementary Table 1 Annotation of identified and quantified proteins from the TMT analysis.

Supplementary Table 2 Differentially expressed proteins in vitrified porcine GV oocytes after IVM.

Supplementary Table 3 Subcellular localization analysis of differentially expressed proteins.

Supplementary Table 4 GO classification analysis of differentially expressed proteins.

Supplementary Table 5 COG/KOG classification analysis of differentially expressed proteins.

Supplementary Table 6 GO enrichment analysis of differentially expressed proteins.

Supplementary Table 7 KEGG pathway enrichment analysis of differentially expressed proteins.
