# Supplementary figures and images for: Proteomic Changes of Porcine Oocytes After Vitrification and Subsequent in vitro Maturation: A Tandem Mass Tag-Based Quantitative Analysis
Source: Front Cell Dev Biol. 2020 Dec 23;8:614577. doi: 10.3389/fcell.2020.614577 (PMC7785821; doi:10.3389/fcell.2020.614577)

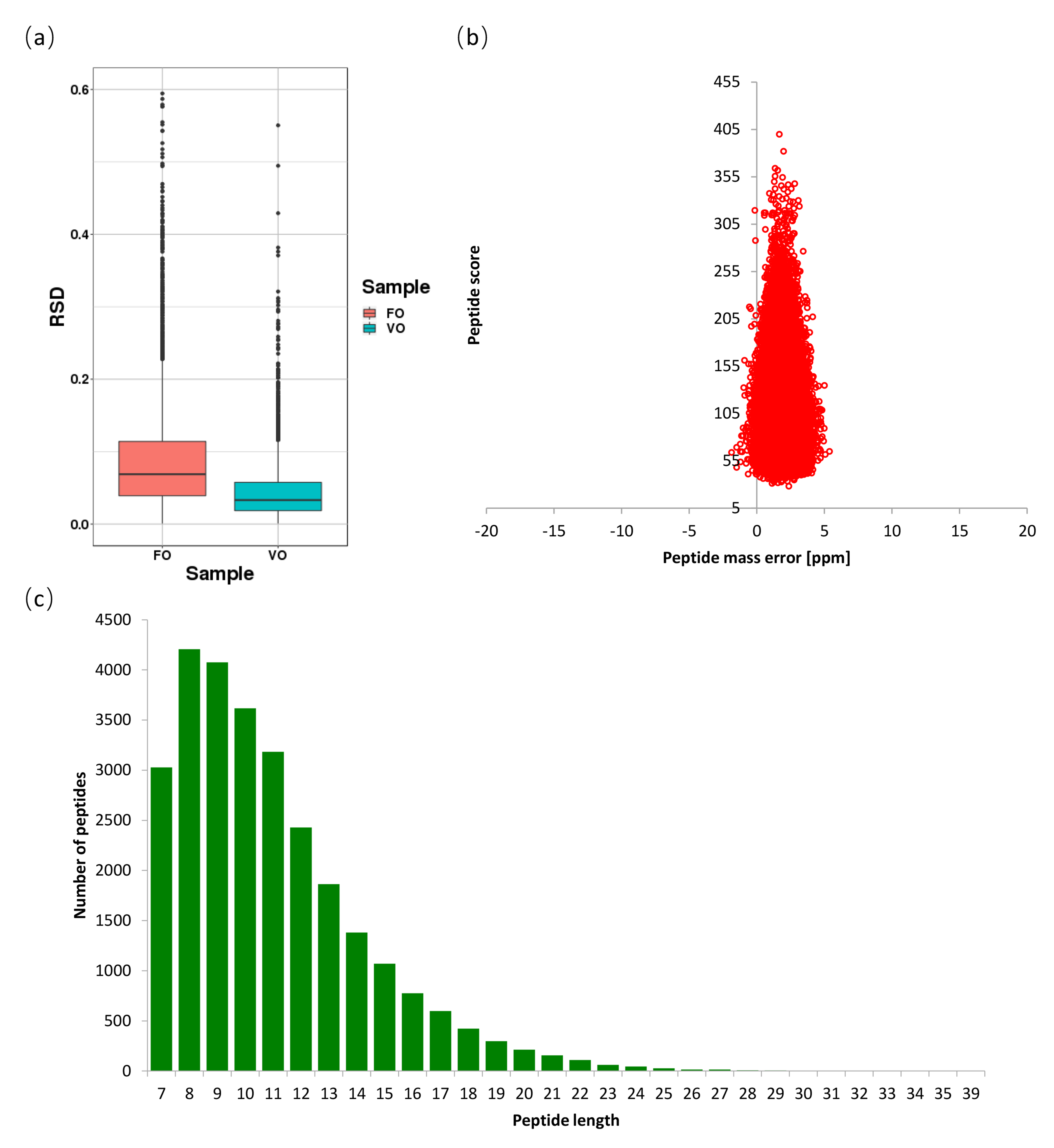

Supplement: Supplementary Figure 1 — Quantitative proteome analysis and quality control validation of mass spectrometry data. (A) Relative standard deviation (RSD) distribution of repeated samples; (B) Average peptide mass error; (C) Length distribution of all identified peptides. [file Image_1.TIF]
